# Use of 5α-reductase inhibitor and delay in prostate cancer diagnosis and treatment

**DOI:** 10.1590/S1677-5538.IBJU.2020.03.02

**Published:** 2020-02-20

**Authors:** Wilson F. S. Busato

**Affiliations:** 1 Universidade do Vale do Itajaí Disciplina de Urologia ItajaíSC Brasil Disciplina de Urologia, Universidade do Vale do Itajaí - Univali, Itajaí, SC, Brasil; 2 Sociedade Brasileira de Urologia Departamento de Uro-Oncologia Rio de JaneiroRJ Brasil Departamento de Uro-Oncologia, Sociedade Brasileira de Urologia - SBU, Rio de Janeiro, RJ, Brasil

## INTRODUCTION

Benign Prostatic Hyperplasia (BPH) and its associated symptomatology affect many men worldwide, the prevalence is over 210 million men and up to 50% of men >50 year-old experience LUTS from BPH (1). BPH has been linked to two factors: age and the presence of testosterone and two drug classes became accepted standard of care 3 decades ago: 5-alpha-reductase inhibitors (5-ARI) and Alpha-blockers (2). The 5-ARI block the intra-prostatic conversion of testosterone to more potent androgen dihydrotestosterone (DHT) and thus reduce the growth effects of androgens on the prostate (3). But 5-ARI also depress serum prostate-specific antigen (PSA) concentrations by approximately 50% (4). In 2003 an article with 18,000 men who randomly were assigned to receive either 5 mg of finasteride or a placebo, the finasteride group had a 25% lower risk of being diagnosed with prostate cancer (PC), but a 68% higher risk of being diagnosed with a high-grade (HG) disease. That study encourage urologists and general practioners (GP) to prescribe finasteride for PC prevention (5). Recently, an article has reopened an important discussion: Can 5-ARI use delay PSA-based PC diagnosis or even increase the risk of a more advanced PC at time of diagnosis when PSA's effect is disregarded (6).

The 5-ARI acts by preventing the intra-cellular conversion of testosterone to DHT. However, this enzyme does not occur in normal or even malignant prostatic epithelial cells, but in stromal cells. Etzioni et al. demonstrated that significant PC (GS>6) rarely occur in prostatic stroma, thus 5-ARI would have little effect on **GS**≥**7** PC, not benefiting in the reduction of these cases (7). But 5-ARI has the ability to reduce PSA production by the prostatic stroma. So, men using 5-ARI need to have their PSA multiplied by factor 2 in the first 2 years of use, by 2.3 between 2 and 7 years and by 2.5 after 7 years of use. Using this correction, screening remains effective in men using 5-ARI (7).

The concern is that these drugs are widely used to treat BPH for long periods of time and are often prescribed by non-urologists. Approximately 90% of PSA screening tests are ordered in primary care by GPs or internists, just 7% were ordered by urologists (8, 9). There is no reliable data in Brazil and it is believed that between 25-30% of PSAs are requested by urologists in private health insurance. But in the public system, serving 70% of the population, the situation is quite different. The official recommendation is that “… upon initial evaluation by the GP, men with suspected PC should be referred to medium-complexity outpatient clinics where the urologist makes the diagnostic investigation.” (10). Finasteride is offered by the public system at no charge. Thus, the suppression of PSA by 5-ARI may not be taken into consideration, delaying the time to refer to the urologist, to biopsy indication and, consequently, worsening oncological results. Men diagnosed with local or regional metastasis from PC have a 5-year survival of 99%; however, men diagnosed with distant disease have a 29% chance of 5-year survival (11).

In the study 80,875 men from the Veterans Affairs Health Care System were evaluated where 8,587 were using 5-ARI at the time of diagnosis of PC. Comparing with those who did not use, these men had longer time between PSA elevation (3.6 years vs. 1.4 years) and biopsy. The mean adjusted PSA at the time of biopsy was significantly higher for 5-ARI users than non-users (13.5 ng/mL vs 6.4 ng/mL). Patients treated with 5-ARI were more likely to have **GS**≥**8** (25.2% vs 17.0%), clinical stage ≥**T3** (4.7% vs 2.9%), positive lymph nodes (3.0% vs 1.7%) and metastatic disease (6.7% vs 2.9%) than non-users. Finally, this study found 5-ARI users to be at greater risk for PC specific mortality than non-users (13% vs 8%), corresponding to an adjusted 39% incremental risk. They concluded that the pre-diagnosis use of 5-ARI was associated with a late diagnosis and worse cancer-specific outcomes in men with PC. Highlight the continuing need to raise awareness about 5-ARI-induced PSA suppression among non-urologists (6).

One of the possible reasons why GPs do not adjust PSA in men taking 5-ARI is the lack of information about it. In fact, the guidelines (AUA, ASCO, UAE, BSU) do not recommend a PSA cutoff in men using 5-ARI to indicate biopsy. It would be important for societies to campaign among all primary care physicians to clarify this issue. Another possible cause is the combined use of 5-ARI with alpha-blockers (combo) that may lead non-urologists to misperceive as monotherapy. On the other hand, urologists need to discuss these issues related to 5-ARI use with patients, explaining that PSA should continue to fall while taking medication and that if there is an increase, the risk of cancer increases by 3x and the risk of a high grade disease increases by 6x (12).

Finally, it is worth leaving Dr. Resnick's words: “… the available data suggest that 5-ARI use is safe. What is not safe, however, is ignoring the PSA suppression associated with 5-ARI therapy.”nfer some protection to the urinary tract function.

## References

[1] 1. Lokeshwar SD, Harper BT, Webb E, Jordan A, Dykes TA, Neal DE Jr, Terris MK, Klaassen Z. Epidemiology and treatment modalities for the management of benign prostatic hyperplasia. Transl Androl Urol. 2019;8:529-39. 10.21037/tau.2019.10.01 PMC6842780 31807429

[2] 2. Lepor H. Medical treatment of benign prostatic hyperplasia. Rev Urol. 2011;13:20-33. PMC3151584 21826125

[3] 3. Goldenberg L, So A, Fleshner N, Rendon R, Drachenberg D, Elhilali M. The role of 5-alpha reductase inhibitors in prostate pathophysiology: Is there an additional advantage to inhibition of type 1 isoenzyme? Can Urol Assoc J. 2009;3(3 Suppl 2):S109-14. 10.5489/cuaj.1114 PMC2698784 19543428

[4] 4. Rittmaster RS. 5alpha-reductase inhibitors in benign prostatic hyperplasia and prostate cancer risk reduction. Best Pract Res Clin Endocrinol Metab. 2008;22:389-402. 10.1016/j.beem.2008.01.016 18471794

[5] 5. Goodman PJ, Tangen CM, Darke AK, Lucia MS, Ford LG, Minasian LM, et al. Long-Term Effects of Finasteride on Prostate Cancer Mortality. N Engl J Med. 2019 Jan 24;380:393-4. 10.1056/NEJMc1809961 30673548

[6] 6. Sarkar RR, Parsons JK, Bryant AK, Ryan ST, Kader AK, McKay RR, et al. Association of Treatment With 5α-Reductase Inhibitors With Time to Diagnosis and Mortality in Prostate Cancer. JAMA Intern Med. 2019;179:812-9. 10.1001/jamainternmed.2019.0280 PMC6503564 31058923

[7] 7. Etzioni RD, Howlader N, Shaw PA, Ankerst DP, Penson DF, Goodman PJ, et al. Long-term effects of finasteride on prostate specific antigen levels: results from the prostate cancer prevention trial. J Urol. 2005;174:877-81. 10.1097/01.ju.0000169255.64518.fb 16093979

[8] 8. Wahl PM, Timmerman J, Mammone V, Blunt AG, Anastassopoulos KP. Trends in prostate cancer screening before and after publication of US Preventive Services Task Force draft guidelines: an analysis of data from a large US Laboratory Service Provider. Value in Health 2016;19:A312-PMD85

[9] 9. Rosenberg M, Crawford D, Newmark J, Steiner M. Use of PSA screening guidelines among primary care physicians. J Urol 2016;195(4S. supplem):MP39-01.

[10] 10. [No Authors] DATASUS/SAI. Detecção Precoce – Informativo. Boletim ano 8, número 2, 2017: <http://www.saude.df.gov.br/wp-conteudo/uploads/2018/03/Informativo-Câncer-dePróstata-2017.pdf> (accessed in December 17, 2019).

[11] 11. Thompson IM, Pauler Ankerst D, Chi C, Goodman PJ, Tangen CM, Lippman SM, et al. Prediction of prostate cancer for patients receiving finasteride: results from the Prostate Cancer Prevention Trial. J Clin Oncol. 2007;25:3076-81. 10.1200/JCO.2006.07.6836 17634486

[12] 12. Liss MA, Thompson IM. Prostate cancer prevention with 5-alpha reductase inhibitors: concepts and controversies. Curr Opin Urol. 2018;28:42-5. 10.1097/MOU.0000000000000464 29095730

